# Advancing and Improving *Preventing Chronic Disease: Public Health Research, Practice, and Policy*

**Published:** 2011-12-15

**Authors:** Samuel F. Posner

Welcome to Volume 9 of *Preventing Chronic Disease: Public Health Research, Practice, and Policy (PCD)*. Each year, I review our progress and articulate our plans for the next year, and I am excited to report that we have accomplished what we set out to do this past year and more. In 2012, we will realize the results of working during the past 2 years to accelerate our production process and more rapidly publish an increasing number of quality submissions. We now also cover a broader range of important and emerging topics and are implementing new e-publishing technologies. *PCD* is moving forward to achieve its mission to facilitate the exchange of information between public health researchers, practitioners, and policy makers to ensure that effective chronic disease prevention and health promotion interventions and policies are readily available to all.

Starting with this article, *PCD* now publishes continuously to respond to the increasingly rapid pace of change in information technology and in public health. Continuous publication means that *PCD* publishes articles as soon as they are ready and posts them every week instead of grouping them into bimonthly issues. We have adopted this publication model in response to an increase in submissions and the availability of new technology. During both 2010 and 2011, *PCD* experienced a 20% increase in the number of submissions from the previous year. We have also seen an improvement in the quality of submissions and a greater breadth of topics covered. Continuous publication allows us to process a higher volume of manuscripts while reducing the time from acceptance to publication. This model also allows *PCD* to make scientific information available as quickly as possible.

*PCD* has also invested in new technologies to streamline our production processes. During 2012, we expect these changes to reduce our time from acceptance to publication by as much as 30%. In implementing these changes, we want to ensure that we maintain the branding and information formats that are familiar and useful to our readers while also increasing *PCD’s* visibility and recognition in the field. We will continue to send out a monthly summary of recently published articles. *PCD’s* cover art has been a hallmark of the publication, and we will continue to produce original images related to our content and make them available not only as downloadable posters but also as e-cards that you can send from the *PCD* homepage (www.cdc.gov/pcd). To receive the most up-to-date information from *PCD*, be sure to subscribe at (http://www.cdc.gov/pcd/subscriptions/index.htm) and receive our RSS feed (http://www.cdc.gov/pcd/rss/pcd.xml).

Since 2004, *PCD* has published special issues and collections of articles on specific topics. We are working on a project to make previously published collections available in a single downloadable file, including the topics of childhood obesity (September 2011), women’s reproductive health (November 2011), and community health measures (July, September, and November 2010). These and other collections are available on our website (www.cdc.gov/pcd). The November 2011 issue of *PCD* includes 2 articles as part of a series that will be published during the next 6 months on the prevalence of chronic diseases among military personnel and veterans. These articles will be available as a collection later this year. We encourage authors to contact us if they are interested in developing a collection of papers on a specific topic (pcdeditor@cdc.gov).

In 2012, we will be introducing other technologies to make our content more readily available to our readers. One of these initiatives is the *PCD* app, which was released in November 2011 for mobile devices such as the iPad. Content from *PCD* is also available through content syndication, which allows Web developers to directly download articles in a format that is ready for posting on local Web pages.

We understand that the way in which readers access and measure the quality of *PCD* is very important to authors. Starting in 2012, *PCD* will be indexed through EBSCO and CrossRef with digital object identifiers (DOI). These 2 indexing services provide readers with greater accessibility to content and are important for authors who want the broadest possible dissemination of their work. We expect that *PCD* will have a Thomson Reuters journal impact factor in the coming year. We recognize the importance of this indicator and are looking forward to having these data available.

All of these changes are being implemented in an uncertain and rapidly changing environment. There has never been question of *PCD* achieving its mission, only how best to make use of the tools and resources available to support it. The accomplishments of *PCD* have only been achievable through partnership and dedication from authors, reviewers, readers, the *PCD* editorial board, Centers for Disease Control and Prevention leadership, and *PCD* staff. We are proud of our accomplishments and the level of recognition *PCD* has achieved during the past 8 years, and we are committed to continually moving forward and improving the quality and content of the journal.

